# Development of an automated region-of-interest-setting method based on a deep neural network for brain perfusion single photon emission computed tomography quantification methods

**DOI:** 10.22038/AOJNMB.2024.75375.1528

**Published:** 2024

**Authors:** Taeko Tomimatsu, Kosuke Yamashita, Takumi Sakata, Ryosuke Kamezaki, Ryuji Ikeda, Shinya Shiraishi, Yoshikazu Uchiyama, Shigeki Ito

**Affiliations:** 1 Graduate School of Health Sciences, Kumamoto University, Japan; 2 Department of Central Radiology Kumamoto University Hospital, Japan; 3 Department of Diagnostic Radiology, Faculty of Life Sciences, Kumamoto University, Japan; 4 Department of Information and Communication Technology, Faculty of Engineering, University of Miyazaki, Japan; 5 Department of Medical Radiation Sciences, Faculty of Life Science, Kumamoto University, Japan

**Keywords:** DCNN, ^ 123^I-IMP, ^ 99m^Tc-ECD, rCBF quantification, ROI setting  A B S T R A C T

## Abstract

**Objective(s):**

A simple noninvasive microsphere (SIMS) method using ^123^I-IMP and an improved brain uptake ratio (IBUR) method using ^99m^Tc-ECD for the quantitative measurement of regional cerebral blood flow have been recently reported. The input functions of these methods were determined using the administered dose, which was obtained by analyzing the time activity curve of the pulmonary artery (PA) for SIMS and the ascending aorta (AAo) for the IBUR methods for dynamic chest images. If the PA and AAo regions of interest (ROIs) can be determined using deep convolutional neural networks (DCNN) for segmentation, the accuracy of these ROI-setting methods can be improved through simple analytical operations to ensure repeatability and reproducibility. The purpose of this study was to develop new PA and AAo-ROI setting methods using a DCNN (DCNN-ROI method).

**Methods:**

A U-Net architecture based on convolutional neural networks was used to determine the PA and AAo candidate regions. Images of 290 patients who underwent ^123^I-IMP RI-angiography and 108 patients who underwent ^99m^Tc-ECD RI-angiography were used. The PA and AAo-ROI results for the DCNN-ROI method were compared to those obtained using manual methods. The counts for the input function on the PA and AAo-ROI were determined by integrating the area under the curve (AUC) counts of the time-activity curve of PA and AAo-ROI, respectively. The effectiveness of the DCNN-ROI method was elucidated through a comparison with the integrated AUC counts of the DCNN-ROI and the manual ROI.

**Results:**

The coincidence ratio for the locations of the PA and AAo-ROI obtained using the DCNN method and that for the manual method was 100%. Strong correlations were observed between the AUC counts using the DCNN and manual methods.

**Conclusion:**

New ROI- setting programs were developed using a deep convolution neural network DCNN to determine the input functions for the SIMS and IBUR methods. The accuracy of these methods is comparable to that of the manual method.

## Introduction

 As tracers that accumulate in brain tissue after passing through the Blood Brain Barrier (BBB), lipid-soluble substances such as N-isopropyl-p-[^123^I] iodoamphetamine (^123^I-IMP) and ^99m^Tc-ethyl cysteinate dimer (^99m^Tc-ECD) are widely used for cerebral blood flow single photon emission computed tomography (SPECT) (1-7). 

 Therefore, various regional cerebral blood flow quantification methods have been developed (8-18).

 A simple noninvasive (without arterial blood sampling) microsphere (SIMS) method using ^123^I-IMP and an improved brain uptake ratio (IBUR) method using ^99m^Tc-ECD as a noninvasive quantitative method have recently been reported (19-24). The input functions of these methods were determined using the administered dose. This was based on the area under the curve (AUC) obtained by analyzing the time activity curve (TAC) for the pulmonary artery (PA) for SIMS and the ascending aorta (AAo) for the IBUR method using dynamic chest images (19-24). 

 An automated region of interest (ROI)-setting program for PA and AAo was developed based on the outcomes of the mathematical and statistical analyses of chest radioisotope (RI) angiograms, respectively (22-24). The coincidence ratio for the locations of the PA and AAo-ROI determined mathematically, and those determined manually was approximately 91−94% (22-24). However, further improvements of approximately 10% are required to use this program in practice. For the SIMS method, the AUC based on the TAC for the PA was the pulmonary inflow (19). For the lung field, the maximum TAC value was normalized to 1. The washout ratio (WR) was calculated, and the product of the lung inflow and WR was used as the input function. The automated determination of WR was accurate, as mean values were used for the lungs filled with ^123^I-IMP (22). Therefore, new methods are required to determine PA and AAo-ROI.

 Deep convolutional neural networks (DCNNs) are based on established algorithms and have been used in the field of medical imaging (25, 26). Additionally, it has proven to be very effective for a variety of applications in the field of medical imaging (27-30). In the field of nuclear medicine, Chen et al. developed a direct method using a DCNN with which attenuation correction was completed solely by the input of SPECT images of cardiovascular blood flow obtained using a semiconductor detector (31). 

 They also developed an indirect method with which a CT attenuation map was derived using a DCNN (31). Hashimoto et al. and Armanious et al. reported highly accurate attenuation correction in brain ^18^FDG-PET images using DCNN-generated CT images (32, 33). In this light, we could use a DCNN to develop a new ROI-setting method that is not affected by various conditions and analyst factors. U-net has been applied to the segmentation of medical images, and it may be highly effective for region extraction because it is very useful for feature extraction and providing positional information (27, 34, 35). In other words, employing U-net is expected to facilitate the extraction of images from specific areas, not only for 3D images but also for 2D dynamic images. In addition, with the capability for specific area extraction, it should also be possible to pinpoint extraction in corresponding regions.

 If the PA and AAo regions can be determined using a DCNN for segmentation, the accuracy of these ROI-setting programs can be improved through simple analytical operations to ensure repeatability and reproducibility. Additionally, these methods facilitate automated ROI- setting that is not only useful in nuclear medicine but also for all imaging examinations.

 This study aimed to develop a new PA- and AAo-ROI-setting program for determining input functions for the SIMS and IBUR methods using a DCNN and to clarify the accuracy of this program by comparing the obtained input function with the manual method.

## Methods

***Ethics statements***

 This study was approved by the Ethics Committee of Medicine at the Kumamoto University for Human Studies (Protocol Number. Advanced 1451, 09/29/2022), and written informed consent was obtained from all patients before the study began. All image data were anonymized, and the study was conducted following the principles of the Declaration of Helsinki and the regulations of the ethics board of each participating institution.

 This was a prospective, comparative, observational study that developed an automated ROI-setting program based on deep neural networks for SPECT images. This study was conducted following strengthening the Reporting of Observational Studies in Epidemiology (STROBE) guidelines.

***Participants***

 The study included 290 patients (male: 197, female: 93, mean age: 59.0 years old) who underwent ^123^I-IMP RI-angiography and microsphere imaging at the same time and 108 patients (male: 74, female: 34, mean age: 70.4) who underwent ^99m^Tc-ECD SPECT and RI-angiography between February 2012 and August 2021 at Kumamoto University Hospital. 

 None of the patients had pulmonary disease. Images obtained from the patients were used as training and validation datasets for the ^123^I-IMP PA-ROI and ^99m^Tc-ECD AAo-ROI methods. The final test dataset for the ^123^I-IMP PA-ROI method comprised the data of 35 patients (23 men, 11 women; mean age: 58.4 years) who underwent ^123^I-IMP RI-angiography and microsphere imaging at Kumamoto University Hospital. For the ^99m^Tc-ECD AAo- ROI method, the final test dataset included the data of 65 patients (30 men, 35 women; mean age: 64.0 years) who underwent ^99m^Tc-ECD RI-angiography and SPECT at Kumamoto University Hospital.

^123^I-IMP chest RI-angiography was performed using a SPECT device (Millennium VG, GE, USA). Imaging was performed at 1 frame/s for 60 s after the ^123^I-IMP bolus injection. The matrix size was 128×128 pixels, and the pixel size was 2.21 mm/pixel (zoom factor: ×2). The collimator was equipped with low energy and high resolution (LEHR). The energy window was set to 159keV±10%.

^99m^Tc-ECD chest RI-angiography was performed using a SPECT device (E-cam, SIE-MENS, Germany) equipped with LEHR collimators. Imaging was performed at 1 fps for 100 s from the start of the ^99m^Tc-ECD bolus injection. The matrix size was 128×128 pixels, and the pixel size was 2.21 mm/pixel (zoom factor: ×2). The energy window was set to 140 keV±10%. The Daemon Research Image Processor manufactured by FUJIFILM RI Pharma and Image J (National Institutes of Health, https://imagej.net/ij/) was used for image processing.

 Standardization of the RI-angiography images

The pixel sizes and positions of the chest RI angiograms differed with the cases because they depended on the gamma camera system used and the patient. The pixel size of all chest RI angiograms was converted to 2.21 mm, which was the smallest size that would prevent the splitting of the voxels using linear interpolation.

***Development of candidate region extraction program using DCNN***

**Figure 1:**
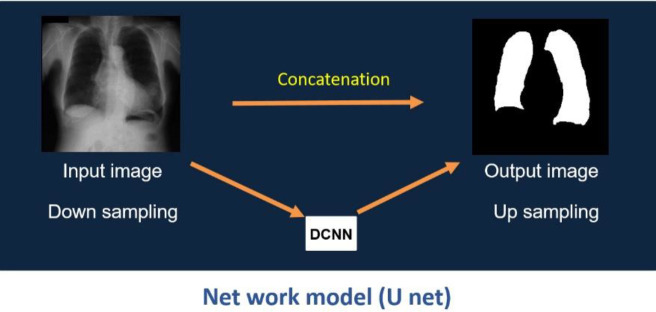
Structure of the U-net

Figure 1 shows the structure of a DCNN (U-net) (27, 34, 35). We applied the ReLU activation function and batch normalization to all convolutional layers, except for the last convolutional layer in the U-net, with depths of five and 13 convolutional layers. Immediately after the last convolutional layer, the squared error was applied to the output layer. The optimization functions for the U-net training were Adam, Alpha=0.001, Beta1=0.9, Beta2=0.999, and Epsilon=1E-8, and the number of iterations was 50. For the learning and validation of the U-net, all samples were randomly divided into six groups, and all cases were evaluated using six-fold cross-validation. The operating environment used was Microsoft Windows 10 Pro, the CPU was an Intel Xeon E5-2623 v3, and the GPU was an NVIDIA Quadro RTX6000. The program was developed using the Sony Neural Network Console.

**Figure 2:**
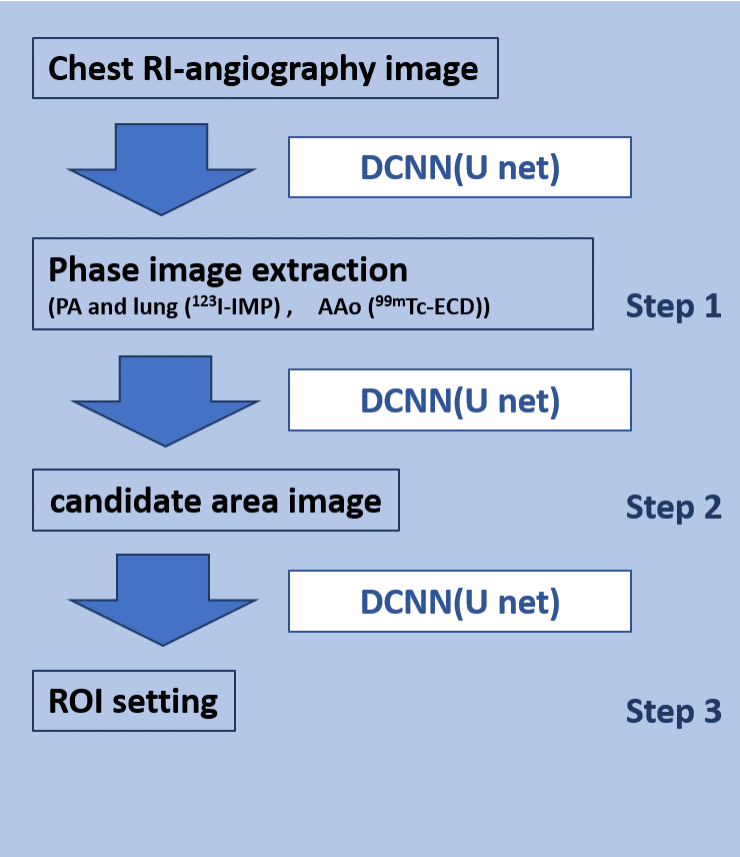
Schematic diagram of DCNN ROI-setting program

Figure 2 shows a schematic of the automated ROI-setting program based on a DCNN. Chest RI-angiography images and manually extracted PA and AAo (^99m^Tc-ECD) blood-phase images were used to train the U-net. For the ROI setting, three researchers extracted the respective optimal ROI areas from the chest RI- angiography image and trained the U-net to learn the average area, which was then used as the final area. Circular ROIs were positioned around the centroid of this area to ensure it did not extend beyond the region.

 Owing to the small sample size of this study, the training accuracy was improved by artificially increasing the amount of data after processing the training images. Data expansion was performed by reversing, rotating in four directions, and translating 10 pixels in eight directions.

***Creation of pulmonary artery (PA) phase and candidate images***

 For the ^123^I-IMP SIMS method, the ROI was set to the PA, and the input function was determined using count analysis of the ROI (19). 

**Figure 3:**
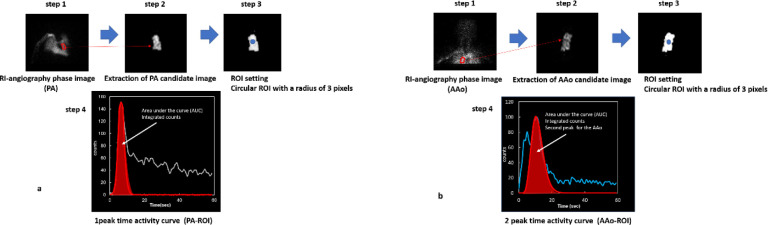
Schematic diagram for the segmentation, ROI setting, area under the curves (AUC), and integrated counts

Figure 3a shows examples of the manually created PA phase images and candidate PA region images. The PA phase image was created by adding two to three frames before and after the peak frame necessary to extract the PA region from the chest RI-angiography image. PA area candidate images were created by marking the PA areas on the PA phase images using ImageJ software. A total of 16,384 learning images were obtained by extending the data from the 256 target cases.

***Creation of ascending aorta (AAo) phase and candidate images (*** ^99m^ ***Tc-ECD)***

 For the ^99m^Tc-ECD IBUR method, an ROI is set on the ascending aorta (AAo), and the input function is determined from the count analysis of the ROI (20, 21). Figure 3b shows examples of the manually generated AAo phase images and candidate AAo region images. The AAo phase image was created by adding two to three frames before and after the peak frame required to extract the ascending aortic region using the normalized thoracic RI-angiography image. The AAo region candidate image was created by marking the AAo region using the ImageJ software. A total of 6804 training images were obtained by extending the data from 108 examples and subtracting the original images.

***ROI-setting***

***PA-ROI***

 First, the PA region phase image and the PA region candidate image were manually created (Figure 3a) (step 1), and the DCNN was trained using the former as the input image and the latter as the training image. Next, the verification image was input, and the PA area candidate image was obtained (Figure 3a) (step 2). For the PA candidate images, areas with counts were identified using the P-tile method, where the pixel values were set to one. Subsequently, binary image (black and white) processing was applied, with 0 or 1 representing the pixel values. Circular ROIs with a radius of three pixels (6.6 mm) were placed at the centroids of these regions (Figure 3a) (step 3).

***AAo-ROI***

 AAo region phase images and AAo region candidate images were created manually using the former as the input image and the latter as the training image for the DCNN training (Figure 3b) (step 1). Next, the verification image was input, and the AAo candidate image was obtained (Figure 3b) (step 2). For the AAo candidate images, areas with counts were identified using the P-tile method, where the pixel values were set to one. Subsequently, binary image (black and white) processing was applied, with 0 or 1 representing the pixel values. The final determination of the PA-ROI location was based on the center of gravity within the candidate region for the pulmonary artery (PA).　Circular ROIs with a radius of three pixels (6.6 mm) were then placed at the centroids of the regions (Figure 3b) (step 3).

 The centroid of the binarized image was determined by calculating the weighted average position of the white areas corresponding to '1,' thus identifying the central position within these white regions (Figure 3a and b) (step 3). 

 To minimize the impact on the centroid ratio, this study employed a fixed Circular ROI and prevented any extension beyond the candidate region.

***Assessment method***

 The accuracy of the DCNN-ROI was clarified by defining the PA and AAo regions on the chest RI-angiography image (red frames in Figsure 3a and b), comparing the centroids of the ROIs set by the DCNN-based ROI-setting program with those determined manually, and evaluating the range of variation between the two methods.

Figure 3a (step 4) shows the time-activity curve (TAC) of the PA-ROI, a single peak of TAC is the target of analysis for PA-ROI (SIMS) (24). 

Figure 3b (step 4) shows the TAC of the AAo-ROI; the second peak of the double-peak TAC is a prerequisite for AAo-ROI (IBUR) (Figure 3b) (22). The counts for the input function on the PA and AAo-ROI were determined by integrating the area under the curve (AUC) counts of the time-activity curve (TAC) of PA and AAo-ROI, respectively. The effectiveness of the DCNN-ROI was elucidated through a comparison with the integrated AUC counts of the DCNN-ROI and the manual ROI.

(**a**) PA-ROI

(**b**) AAo-ROI

***Statistical analysis***

 All data are expressed as mean ± standard deviation (SD) and were statistically analyzed by t-test using MedCalc Statistical Software version 20.115 (Med Calc. Software Ltd., Ostend, Belgium; https://www.medcalc.org, 2020). Statistical significance was set at p<0.01. The Altman analysis was used to assess the agreement between the manual and DCNN-based ROI-setting methods.

## Results

***Comparison of ROI positions***

***PA-ROI***

**Figure 4:**
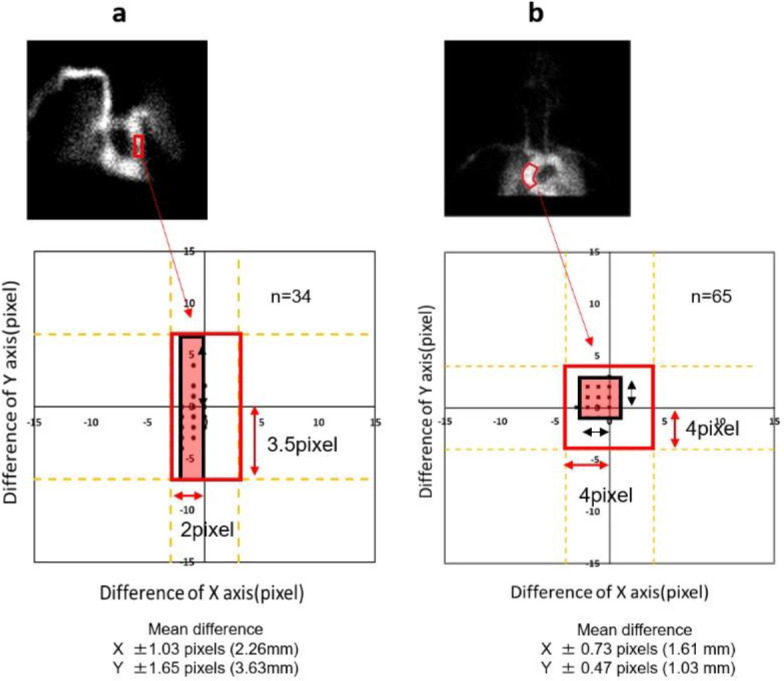
Comparison of ROI location (**a**) PA (**b**) AAo

 The manual PA-ROI determined by histogram analysis of the PA images was used as the reference. Figure 4a shows the distribution of the differences between the x- and y-axis directions of the DCNN PA-ROI. When the match condition was ±2 pixels for the x-axis and ±3.5 pixels (8.5 mm) for the y-axis, which is the range matching the size of the PA, all 35 cases matched. The coincidence ratio for the automated and manual methods was 100% (34/34).

***AAo-ROI***

Figure 4b shows the distribution of the difference between the x- and y-axis directions of the DCNN-based AAo-ROI based on the manual ROI point of the AAo image. The mean difference between the manual and DCNN-based AAo-ROI was ±0.73 pixels (1.61 mm) for the x-axis and ±0.47 pixels (1.03 mm) for the y-axis. When the match condition was set to ±4 pixels (8.8 mm) for the x-axis and ±4 pixels (8.8 mm) for the y-axis, which is the range matching the size of the PA, all 65 cases were matched. The coincidence ratio for the automated and manual methods was 100% (65/65).

***Clinical case image***

**Figure 5:**
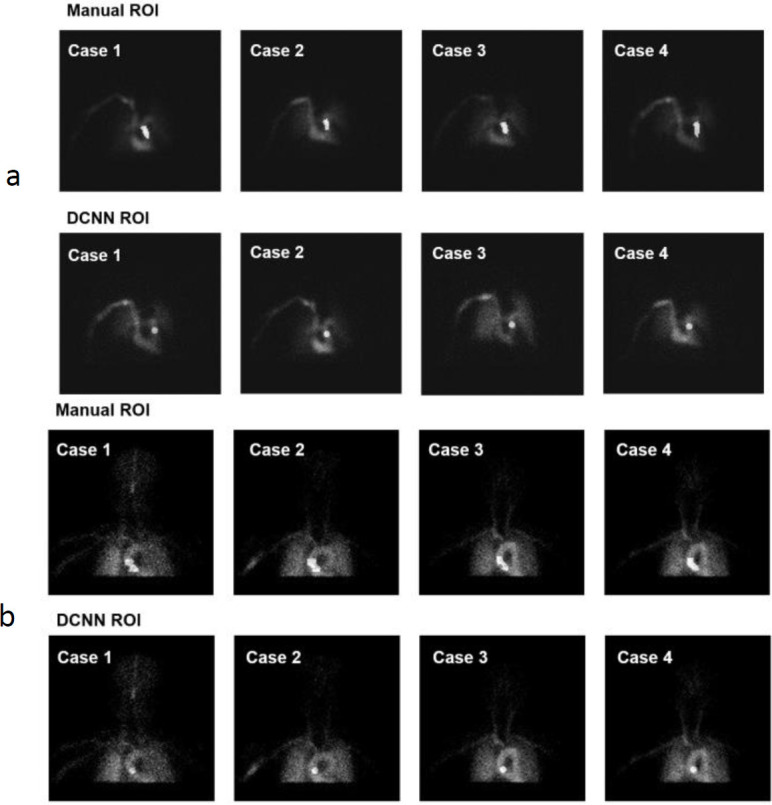
Comparison of ROI images. (**a**) Image extraction and PA ROI-setting for the SIMS method (^123^I-IMP PA) (**b**) Image extraction and AAo ROI-setting with the IBUR method (^99m^Tc-ECD AAo)

 Manual ROIs in Figure 5 represent candidate regions for ROI configuration (corresponding to step 2 in Figure 3a and 3b). The DCNN-ROIs are shown for PA and AAo -ROI configurations, respectively. In all cases, the DCNN-ROIs were set within the boundaries of the manual ROIs.

***Comparative evaluation with manual method***

**Figure 6:**
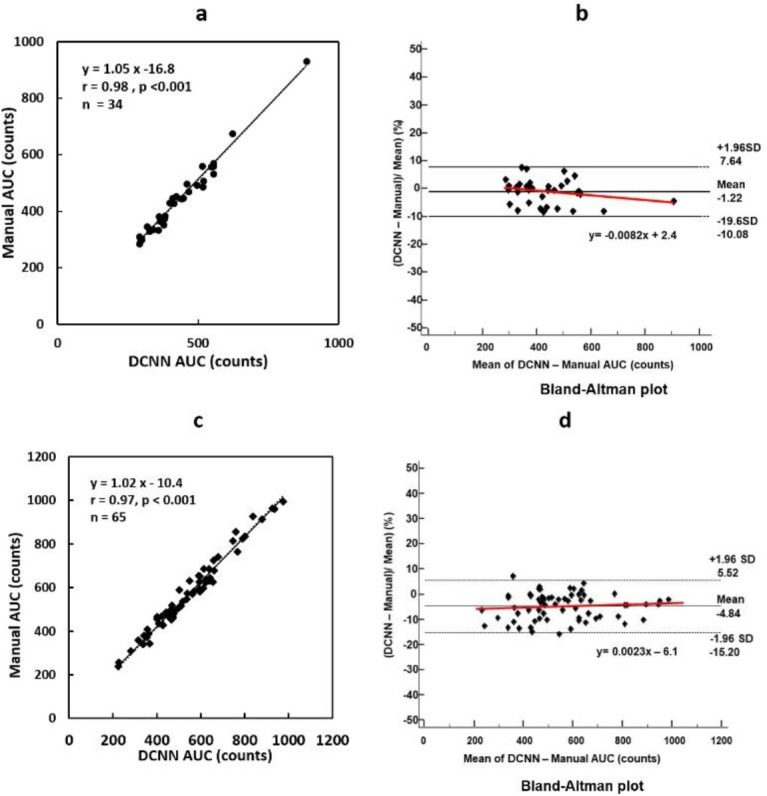
Relationships between the AUC values for the DCNN and manual ROI methods

Figure 6a shows the relationship between the DCNN AUC PA(x) and manual AUC PA (y). The relationship between the two is expressed as y=1.05x - 16.8, and the correlation coefficient is r=0.98 (p<0.01), showing a very good correlation. In the Bland-Altman analysis, the mean difference between the AUCs for the DCNN and manual methods was -1.22%, as shown in Figure 6b. The proportional regression equation was y=-0.0082x +2.4, and very small negative fixed errors were observed,

as shown in Figure 6b. 

 The relationship between the DCNN AAo AUC (x) and manual AUC (y) was expressed as y=1.02x - 10.4, and the correlation coefficient was r=0.97 (p<0.01), indicating a very good correlation as shown in Figure 6c. The mean difference between the AUCs for the DCNN and manual methods was 4.84%. The proportional regression equation was y=0.0023x -6.1, and very small positive fixed errors were observed, as shown in Figure 6d.

(**a**) Relationship between the AUC values for the DCNN and manual ROI methods for PA

(**b**) Bland-Altman plot between the DCNN and manual AUC values for the PA-ROI

(**c**) Relationship between the AUC values for the DCNN and manual ROI methods for AAo

(**d**) Bland-Altman plot between the DCNN and manual AUC values for the AAo-ROI

## Discussion

 We developed an automated ROI-setting program that determines a new input function using a DCNN and the SIMS and IBUR methods (19, 20) based on brain perfusion SPECT image analysis developed for the field of nuclear medicine.

 For the SIMS method using ^123^I-IMP, it is necessary to set two ROIs in the PA and lung to determine the input function (19, 23, 24). The automated WR determination program is accurate without any modification, as the WR is obtained using the mean uptake counts for the lungs filled with ^123^I-IMP (22). Therefore, new methods for PA and AAo-ROI determination are required. For the IBUR method using ^99m^Tc-ECD, it is necessary to set an ROI in the AAo when determining the input function (20-22). 

 Therefore, we developed an automated ROI-setting program for PA and AAo. For this program, phase images of the PA and AAo were obtained from chest RI-angiography by frame addition, with the peak frame necessary for extracting each region as the center. After learning the segmentation to generate the candidate from the phase region image to the DCNN, binarization was performed on the candidate region images generated by inputting the verification images into the DCNN, and the ROI was set at the center of gravity of each region. We verified the accuracy of the DCNN method by comparing its ROI with that set by the manual method and the accompanying AUCs. 

Table 1 shows a comparison of the mean difference with respect to the manual method between the DCNN and Auto methods. The DCNN PA-ROI was set within the specified PA range of the PA for all 34 visually evaluated cases. From the evaluation of the difference from the center of the PA ROI manually set by three research staff, the ROI was set within the allowable range for all cases, even when the allowable range was deviated by ±1 (2.2 mm) pixels on the a-axis and ±3.5 (7.7 mm) pixels on the y-axis from the size of the PA (Figure 4a). 

 This range was consistent with that reported by Yamashita et al. (24). Furthermore, the coincidence ratio was 100% for the DCNN-ROI, compared with the 94% reported by Yamashita et al. Therefore, the DCNN-ROI method is comparable to the automated ROI method.

**Table 1:** Comparison of the mean difference with respect to the manual method between the DCNN and auto method

		P A R OI for the^123^I-IMP SIMS		AA0 R OI for ^99m^TC-E C D IBU R	
ROI setting method		DCNN	Auto ^a^	DCNN	Auto ^b^
Mean difference to the manual method (m m)	X Direction	±2.2	±2.2	±1.0	±8.6
	Y Direction	±7.7	±7.7	±1.6	±6.4
Coincidence ratio (%)		100	94	100	98

 As shown in Figure 5a, the DCNN PA and manual AUCs showed a very good correlation (r=0.97). In addition, the average error was -1.22%, indicating good agreement. Yamashita et al. reported that the ROI position and AUC for the automated ROI-setting method using phase analysis showed good correlations (r=0.91) with those for the manual method (24). Therefore, the DCNN-ROI method may be comparable or superior to the automated ROI method proposed by Yamashita et al (24).

 For the automated ROI-setting in this study, the ROI was set within a specified range for all 65 visually evaluated cases. The evaluation of the difference from the center of the PA-ROI manually set by three research staff showed that the ROI was set within the allowable range for all cases, even when the allowable range deviated by ±0.73 (1.6 mm) pixels on the x-axis and ±0.47 (1.03 mm) pixels on the y-axis from the size of the pulmonary artery (Figure 4b). 

 Masunaga et al. reported a difference between the ROI locations obtained by the automated and manual methods for the AAo-ROI. They also reported that the mean difference between the ROI locations was 2.9 pixels (6.4 mm) on the x-axis and 3.9 pixels (8.6 mm) on the y-axis (22). 

 Furthermore, the coincidence ratio was 100% for the DCNN-ROI method relative to the 98% reported by Masunaga et al. (22). The DCNN-ROI method matched the manual method within a narrower range of approximately 25% of that reported by Masunaga (22). This indicates that the variability in the AUC due to TAC analysis may be reduced. Therefore, the DCNN-ROI method was superior to the automated ROI method proposed by Masunaga et al. (22).

 As shown in Fig 5b, the DCNN AAo AUC counts calculated by this program and the manual AUC counts demonstrated a very good correlation (r=0.97). Masunaga et al. reported that the ROI position and AUC using phase analysis in the automatic ROI setting method had a good correlation (r=0.99) with those in the manual method (22). Therefore, the DCNN-ROI method may be comparable to the automatic ROI method by Masunaga et al. (22).

 In the Bland-Altman analysis, minimal fixed errors were observed between the AAo and PA AUCs for the DCNN and manual methods, as shown in Figure 6b and d. However, given the negligible magnitude of these errors, it is believed that they do not introduce any significant systematic bias.

 In this study, the DCNN-ROI method was compared with the manual method and the DCNN method from the accuracy viewpoint of the ROI-setting position and AUC counts; the usefulness of the DCNN-ROI method was proven in all cases. When generating a regional phase image using the conventional automatic method based on phase analysis, there is a high possibility that extraction would be difficult due to the subtraction process in the phase analysis (22). In our method, the phase analysis is only performed during frame addition to create regional phase images, eliminating the effect of bolus injection properties and allowing ROI-setting even for cases that were difficult to process with the conventional automatic method. In addition, in terms of the time required for ROI-setting in this program, it takes 1 to 2 days for U-net to learn images for the first time, but thereafter, the region candidate image can be generated using the trained U-net and the automatic ROI-setting can be done within an even shorter period of time than in the automatic method.

 Based on the above, since this program also ensures reproducibility, compared to noninvasive brain perfusion quantification methods, the DCNN-ROI method ensures high reproducibility and simplification of analysis operations, thereby eliminating analytical differences between institutions. We believe this method is clinically useful, considering that this program can accommodate patients with heart disease and problems depending on the drug administration side, which the conventional automatic method had difficulty addressing. Specifically, it is believed that the accuracy of DCNN-ROI will significantly improve by increasing the number of study cases. 

 Therefore, enhancing the precision of the DCNN-ROI method requires an expansion of the training dataset. Conducting clinical trials involving the same patients is essential for comparing the conventional method with the DCNN-ROI method, thereby validating their respective merits and drawbacks and elucidating their distinct characteristics.

 Three researchers extracted the respective optimal ROI areas from the chest RI-angiography image and trained the U-net to learn the average area. The utility of this program could be further demonstrated by requesting the analysis of manual ROIs by operators not involved in the DCNN's automated ROI analysis program.

 In the PA-ROI setting by the DCNN-ROI method, it is difficult to separate the descending vena cava, right heart system, and PA in cases where they are located close to each other, which may negatively affect the accuracy of ROI. Therefore, there is currently no other choice but to perform manual procedures for cases with peculiar anatomical locations, and future countermeasures are necessary to address these cases.

 In this study, 1-peak TAC is the target of analysis for PA ROI (SIMS) (Figure 3a), and 2-peak TAC is a prerequisite for AAo-ROI (IBUR) (Figure 3b). In particular, the injection rate of radiopharmaceuticals is very important. It should be recognized that extremely slow injection rates are unsuitable for quantitative analysis. For this reason, this study excluded data for slow injection rates from the analysis.

 This study presents results from a single institution and devices. Hence, it is necessary for future research to conduct the validation using different equipment in various facilities.

## Conclusion

 New ROI-setting programs were developed using a deep convolution neural net-work DCNN to determine the input functions for the SIMS and IBUR methods. The accuracy of this method was comparable to that of manual ROI methods that utilize mathematical phase image analysis.

## Data Availability

The datasets generated and/or analyzed during the current study are available from the corresponding author upon reasonable request.
